# The diagnosis of asthma. Can physiological tests of small airways function help?

**DOI:** 10.1177/14799731211053332

**Published:** 2021-10-23

**Authors:** Mohammed A Almeshari, James Stockley, Elizabeth Sapey

**Affiliations:** 1 Rehabilitation Health Sciences Department, College of Applied Medical Sciences, King Saud University, Riyadh, Saudi Arabia; 2 Institute of Inflammation and Ageing, 1724 University of Birmingham, Birmingham, UK; 3 Department of Lung Function and Sleep, 1732 University Hospitals Birmingham NHS Foundation Trust, Birmingham, UK; 4 Institute of Inflammation and Ageing, 1724 University of Birmingham, Birmingham, UK

**Keywords:** Asthma, small airways function, diagnosis, spirometry, oscillometry

## Abstract

Asthma is a common, chronic, and heterogeneous disease with a global impact and substantial economic costs. It is also associated with significant mortality and morbidity and the burden of undiagnosed asthma is significant. Asthma can be difficult to diagnose as there is no gold standard test and, while spirometry is central in diagnosing asthma, it may not be sufficient to confirm or exclude the diagnosis. The most commonly reported spirometric measures (forced expiratory volume in one second (FEV_1_) and forced vital capacity assess function in the larger airways. However, small airway dysfunction is highly prevalent in asthma and some studies suggest small airway involvement is one of the earliest disease manifestations. Moreover, there are new inhaled therapies with ultrafine particles that are specifically designed to target the small airways. Potentially, tests of small airways may more accurately diagnose early or mild asthma and assess the response to treatment than spirometry. Furthermore, some assessment techniques do not rely on forced ventilatory manoeuvres and may, therefore, be easier for certain groups to perform. This review discusses the current evidence of small airways tests in asthma and future research that may be needed to further assess their utility.

## Background

Asthma is a highly heterogeneous, chronic respiratory disease with variations in inflammatory processes, clinical course, severity and response to treatment. Inflammation of the airways leads to both clinical symptoms such as chest tightness, wheezing, coughing (classically worse at night or early morning^1^) and variable expiratory airflow limitation that changes with time and intensity, becoming fixed in a proportion of cases.

Symptomatology, exacerbations, airways inflammation and airway remodelling can be reduced with an appropriate diagnosis and treatment plan. However, there is still a significant burden of undiagnosed, hence untreated asthma in both children and adults.^2^ Case-finding studies suggest under-diagnosis is common, with one large study describing undiagnosed asthma in 50% of all cases.^3^ Confirming the diagnosis is also associated with diagnostic delay, with one study describing a median delay between meeting symptom-based criteria and a physician diagnosis of 1.7 years.^4^ Undiagnosed asthma and diagnostic delay are critical, as both are linked to poor clinical outcomes.^5,6^

Misdiagnosis is also common, with studies describing approximately 30% of patients who are diagnosed with asthma having no objective evidence of asthma when tested more thoroughly.^7,8^ Over-diagnosis is also problematic, as potentially alternative diagnoses may be missed and patients might take long term therapy which is not required.

Some of this diagnostic uncertainty reflects the fact that there is no gold standard test to diagnose asthma. A diagnosis is based on suggestive symptoms and a detailed medical history and examination, as well as objective measures, most commonly spirometry with reversibility and serial peak flows.^9^ However, symptoms can be variable and intermittent as well as being common across a variety of respiratory and non-respiratory medical conditions.

Although spirometry is used to objectively assess asthma, the sensitivity of spirometry to diagnose asthma has been quoted to be as low as 29% against a reference standard of bronchial provocation test.^10^ Normal spirometry does not exclude asthma, as airflow obstruction can be transient, manifesting only at certain times of the day or in response to allergy triggers.^11^ Abnormal results do not diagnose asthma, as airflow obstruction may be observed in patients with other diseases such as Chronic Obstructive Pulmonary Disease (COPD).^10,12^

Performing spirometry can be challenging as measurements require significant patient effort and an ability to follow the instructions of healthcare specialists.^10,13^ Exhaling forcefully through a mouthpiece is not a natural breathing manoeuvre, which can make it challenging to obtain accurate results.^14^ Results are interpreted in comparison with predicted values based on height, age, sex and race. However, the ‘normal’ reference ranges are wide and differentiating between health and disease is not always straightforward. Furthermore, the observable differences in lung size across ethnic groups are not all represented sufficiently in reference populations,^15^ just as the extremes of age are under-represented.

Traditionally reported spirometric parameters such as the forced expiratory volume in one second (FEV_1_), the forced vital capacity (FVC) and FEV_1_/FVC may not reflect dysfunction in the small airways.^16^ Indeed, asthma was classically considered a disease of the large airways but there has been an increasing awareness that the small airways (defined as bronchial passages less than 2 mm in diameter) are also affected. Autopsy specimens from fatal cases of asthma have demonstrated inflammation in both the small and large airways with no definitive differences in the composition of inflammation at either site.^17^ Small airways dysfunction (SAD) has been described in children with mild or intermittent asthma symptoms with a normal FEV_1_, suggesting small airways involvement is an early manifestation of the disease.^18^ In a study of transbronchial biopsies, the inflammatory cell infiltrate in the small airways was greater than that in the medium and large airways in patients with poorly controlled asthma symptoms.^19^ Furthermore, there are now inhaled treatments with extra-fine particles that can target the small airways, leading to increased symptomatic benefit and improved clinical outcomes for asthma patients.^20^ All of these studies suggest the small airways might be important in asthma and an important therapeutic target. In theory, physiological assessment of small airways function could also provide the means to diagnose asthma with greater certainty and monitor response to treatment.

In this review, the pathological basis for small airways involvement in asthma will be discussed alongside the current tools available to assess small airways function. We will consider the current evidence to support whether tests of small airways could diagnose asthma or the response to treatments, which can now target small airway function.

### The small airways and the evidence for their involvement in asthma

Studies suggest that lungs with a volume of 5 L contain approximately 30,000 small airways and that most small airways are 0.51 mm–1.0 mm in diameter.^21^ These airways account for more than 98% of the cross-sectional area of the lung, terminate within the alveolar sacs, contain no cartilage to support their structure and are, therefore, more prone to collapsing.^22^ However, the large cross-sectional surface area of the small airways also means that they only account for 10% of the total airway resistance.^23^ This led to the small airways being described as the ‘quiet zone’ as extensive small airways disease can be present with little abnormality in conventional pulmonary function tests, which are insensitive to small airways function.^24^

There is increasing evidence that small airways involvement is present in asthma and that it is associated with both worse symptoms and poorer clinical outcomes.^22,25^ Pathological post-mortem studies have demonstrated inflammation and airway remodelling in the small airways of asthmatic patients, especially those of an eosinophilic phenotype, which traditionally can often be reduced with appropriate treatment.^26,27^ Biopsy studies in asthma have also confirmed the presence of small airways inflammation and alveolar eosinophils, the burden of which correlated with lung function decline.^28^ An increase in airway smooth muscle mass, mucus plugging and goblet cell hyperplasia have all been described in the large and small airways in asthma^29^ but there appear to be differences in the distribution of inflammation. Post-mortem studies in fatal asthma suggest that inflammation is mainly contained within the inner wall of the large airways. In contrast, inflammation tends to occur in the outer airway wall of the small airways,^27^ extending to the perivascular region of the pulmonary arteries and the peribronchiolar alveoli.^30^

Children with asthma have been shown to have greater disease involvement in the peripheral as opposed to the central airways,^31^ and SAD has been described in children with mild or intermittent asthma symptoms, often in the absence of an abnormal FEV_1_^18^. This might lead to the assumption that SAD is an early feature of asthma. However, SAD is not universal in asthma, and it appears to affect only a subset of patients. In the previously discussed study of small airways function in children, only one third had abnormalities in tests of small airways function.^18^ A systematic review conducted in 2016 suggested that SAD could be measured in approximately 50–60% of patients with asthma and that SAD could occur in asthma patients without any evidence of larger airway obstruction.^22^ A multinational study called AssessmenT of smalL Airways involvemeNT In aSthma (ATLANTIS) in 2019 assessed the presence of SAD in 773 adults with asthma using multiple tests of small airway function.^32^ In this group, SAD was also common but, crucially, different SAD techniques and devices altered the detected prevalence of SAD, suggesting that different tests identified different groups of patients. This highlights the critical nature of test selection when comparing studies (discussed in more detail later).

### Small airways dysfunction as a phenotype of asthma

A phenotype can be defined as any observable clinical characteristic or trait of a disease that stratifies its presentation, outcomes or response to treatment, without any implication of a mechanism.^33^ Asthma is now stratified into clinical phenotypes, with the aim of providing treatment pathways based on the likelihood of response.^34^ Cluster analyses have identified asthma phenotypes using several different features, including the age of symptom onset, the presence of atopy, the severity of airways obstruction, the presence of co-morbidities such as obesity and the requirement and response to medication.^35^ One notable analysis of 439 patients described several asthma phenotypes: early-onset atopic asthma, obesity-associated asthma, non-eosinophilic asthma, benign asthma, early symptom predominant asthma and inflammation predominant asthma, with treatment recommendations for each phenotype.^36^ Several trials have been proposed to classify phenotypes of asthma by observing the response to triggers or clinical characteristics.^37^

Given the SAD is only present in a proportion of asthma patients, it is possible that this too could be considered a phenotype. Studies comparing asthma patients with and without small airways disease (measured using physiological tests) have described clinical differences between the groups. These include a reduced FEV_1_ in combination with a history of smoking, raised blood eosinophils and poorly controlled asthma.^38^ Bronchial hyper-responsiveness (BHR) has also been shown to be a feature of those with SAD. In one study of 94 patients with asthma, those with small airways obstruction had more severe BHR as well as lower FEV_1_, FVC and FEV_1_/FVC values and higher levels of reversibility.^39^ In the same study, patients with small airways obstruction using inhaled corticosteroids (ICS) had a significantly higher daily dose of ICS than patients without small airways obstruction (800 vs 500 μg per day beclomethasone dipropionate equivalent).

There are also specific therapies that may be more beneficial to those with SAD, particularly inhalers with extra and ultrafine particles that are more likely to reach the small airways. Inhaler devices that have been routinely used in clinical practice may not effectively deliver ICS into small airways. Conventional aerosol-generating devices such as small-volume nebulizers (SVN) or pressurised metered-dose inhalers (pMDI) generate fine, aerosolized particles with a mass mean aerodynamic diameter (MMAD) of 2–5 μm. Although other factors contribute to drug deposition in the lungs (including inhaler technique and flow rates), particles of this size may not efficiently reach small airways compared to extra-fine inhalers (1–2 μm).^40,41^

Particle size is a major determinant in the deposition and distribution of inhaled drug within the lungs. In a study of 12 asthmatic subjects, the lung distribution of inhaled technetium-99m-labelled monodisperse albuterol aerosols was assessed by planar gamma-scintigraphy. Here, smaller particles achieved greater total lung deposition but bronchodilation (assessed by FEV_1_ and MMEF) was greatest with the larger particle size.^42^ Ciclesonide is an extra-fine particle ICS (with a mass median aerodynamic diameter (MMAD) of 1.0 μm) that shows both high overall lung deposition and peripheral lung distribution in healthy volunteers and patients with asthma.^43,44^ Moreover, ciclesonide has been associated with improved asthma outcomes in patients, including a reduction in exacerbation frequency, better asthma control and improved tests of small airways function.^20,45^ This highlights the need of more research with an emphasis on small airways response in asthmatic subjects.

There has been a move to develop extra-fine particles (defined as particles less than 2 μm in MMAD) for inhalation to address this limitation. These particles have shown higher deposition rates in the lung periphery and a better response to treatment, especially when patients have evidence of SAD.^46^ Currently, the EU Clinical Trials Registry 16^47^ and the USA Clinical Trials Registry collectively report 151 studies on treating small airways in asthma,^48^ highlighting the interest in this area.

Endotypes are defined as subtypes of a disease or condition which share pathophysiological feature at the molecular and/or cellular level.^49^ A number of asthma endotypes have been proposed but currently it is unclear if SAD is associated with a separate endotype, or if SAD is present across several endotypes.^50^ The limited studies to date have not found a ‘small airways specific’ inflammatory endotype but, in common with the larger airways, have described higher numbers of activated eosinophils in the small airways of patients with asthma.^51^ In addition, alveolar inflammation has been specifically implicated in nocturnal symptoms, perhaps reflecting a symptom-based phenotype.^52^

In summary, current evidence suggests that small airways involvement could be a phenotype of asthma, which may be associated with clinical traits and a treatment response. Evidence to support SAD reflecting a specific endotype is currently lacking, but SAD has been associated with a predominance of eosinophilic inflammation.

## The diagnostic challenge of asthma; can tests of small airways help?

The lack of a gold standard test makes diagnosing asthma challenging in some circumstances and, although most guidelines include similar steps to confirm a diagnosis, there are some differences. In Table 1, the current objective tests used by various guidelines are listed alongside their limitations. Of note, current guidelines do not include tests of small airways function in the diagnostic process. This may represent a missed opportunity given that patients with SAD and asthma tend to have poorer symptom control and that there are now treatments available to target this dysfunction. Studies have emphasised that the uptake and implementation of asthma guidelines remain suboptimal globally,^53–55^ with some suggestion that the lack of objective measures to diagnose asthma (rather than support a diagnosis) are hindering patient care. Therefore, improving diagnostic criteria could improve patient outcomes. Perhaps tests of small airways could offer an opportunity to improve the diagnosis of asthma and identify patients who may benefit most from specific therapies, such as ultrafine inhaled medicines. There are several methodologies that can assess small airways function. Unfortunately, there is currently no agreed gold standard and specific small airways tests are often selected over others with no clear rationale. The most commonly used tests of small airways function are discussed below.

**Table 1.** Summary of objective tests used in diagnosing asthma by various organization.

Objective test	Recommended by which organization	Recognized limitations of test
Spirometry	NICE^92^	FEV_1_ limitation may not be a marker for asthma as it is found in other obstruction diseases.^93^ The test is effort-dependent
Reversibility	GINA^94^, BTS/SIGN,^95^ SINA,^93^ NICE, ATS/ERS (severe)	Not only asthmatic patients have reversibility.^96^ Effort-dependent
Peak flow monitoring	GINA, BTS/SIGN, SINA, NICE	Effort-dependent. Different PEF metres may have different results
Bronchial challenge	GINA, BTS/SIGN, SINA	Not as widely available, can be time consuming
DL_CO_	Not recommended, only in severe asthma (ATS/ERS), and not all cases	
Biomarkers of inflammation in sputum (eosinophilic and neutrophilic)	BTS/SIGN, SINA	Not all patients produce sputum No “normal” ranges for cytokines. May be present with other diseases.^94^ Needs technical expertise and time consuming^97^

NICE: national institute of clinical excellence UK; FEV1: forced expiratory volume in the first second; GINA: global initiative for asthma; BTS/SIGN: british thoracic society/scottish intercollegiate guidelines network; SINA: Saudi initiative for asthma; ATS/ERS: American thoracic society/European respiratory society; DL_CO:_ diffusing capacity for carbon monoxide.

### Physiological techniques to assess small airways function

#### Spirometry

Spirometry can provide information about small airways function, including measures of the forced expiratory flow when 50% (FEF_50_) and 75% (FEF_75_) of FVC has been expired as well as the average flow over 25–75% range (FEF_25-75_). The FEF_25-75_ is also referred to as the maximal mid-expiratory flow (MMEF). This measurement has been used to assess small airways limitation in certain patient groups.^56^ However, the FVC can affect test results and it is recommended that MMEF is corrected for FVC, particularly in bronchodilator response testing.^57,58^ As MMEF is a highly variable measure, current spirometry guidelines only utilise FEV_1_ and FVC as the clinically important measurements.^59^ In 2014, a large, retrospective study suggested there was little value in reporting MMEF where FEV_1_ and FVC was available,^60^ which questioned the use of this test in clinical care. However, the participants included in this study were extremely heterogeneous, and not representative of a specific disease population, thereby limiting the results of the analysis. Studies in highly selected populations show that MMEF can be a useful physiological marker of small airways function that relates to different aspects of disease and its progression.^56,61^

### MMEF in asthma

The MMEF has been used to help exclude cough variant asthma from chronic cough of other causes^62^ and has been found to be abnormal in patients with mild asthma and bronchodilator reversibility.^39^ Compared to the FEV_1_, the percentage predicted of MMEF has consistently been lower in multiple groups with asthma, despite a normal or near-normal percentage predicted FEV_1_^63^. Nevertheless, high variability and poor reproducibly are the major drawbacks of using MMEF in diagnosis.

### Oscillometry

To overcome the limitations of maximal forced breathing manoeuvres, other tests that do not rely on such effort-dependent manoeuvres should be considered. These include forced oscillation techniques (FOT) and impulse oscillometry (IOS). The FOT was first developed in the 1950s and uses sinusoidal pressure oscillations.^64,65^ The IOS uses the same concept but uses square wave pressure oscillation (‘pulses’) of multiple frequencies of oscillation at the same time. Both FOT and IOS are easy to perform, but result in complex measurements, describing lung impedance (Zrs; defined as the spectral relationship between volume and pressure) from which lung reactance (Xrs; defined as the amount of airway recoil against the oscillating pressure wave) and resistance (Rrs; defined as the amount of energy required for the oscillating pressure wave to move through the airways) are derived.^66^

**Figure 1.**
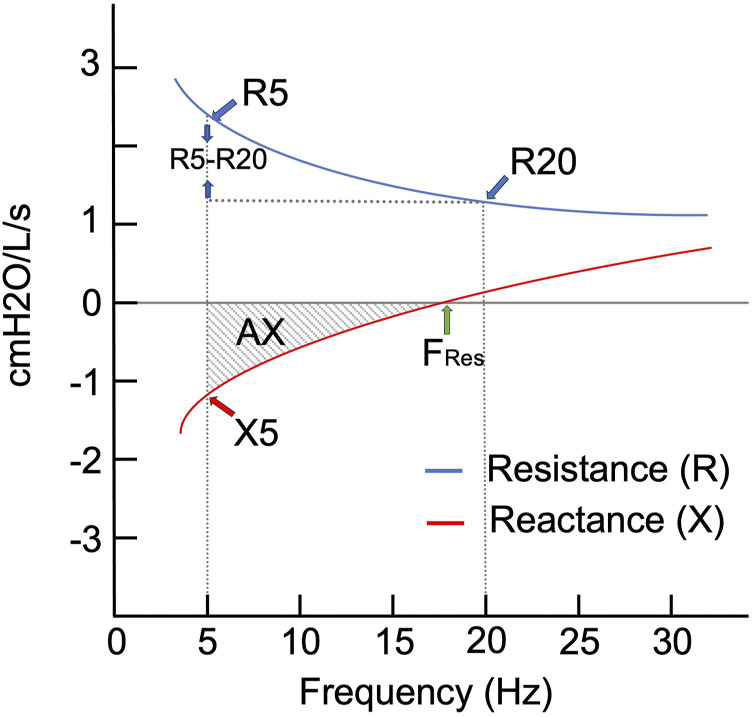
Illustration of oscillometry technique indices locations in resistance and reactance curves. Legend: The red line is the reactance line, and oscillation at 5 Hz is (X5). When reactance pressure reaches 0, this is the point of resonant frequency (Fres). The area under the curve between X5 and Fres is the area of reactance (AX). The blue line is the resistance line, and resistance at 5 Hz is the total lung resistance (R5). Resistance at 20 Hz is the large airways resistance (R20). The difference in resistance between R5 and R20 is considered as the small airways resistance (R5-R20).

All oscillometry devices generate oscillating sound waves between 3 and 35 Hz, with higher frequencies travelling shorter distances (measuring larger airways) and lower frequencies travelling further (measuring ‘total’ airway function). The use of multiple frequencies enables the assessment of most of the airway tree^65,67^ and although oscillometry is primarily used to assess SAD, it can also be used to assess the large airways.^68^ Higher resistance within the airways (for example, in the presence of bronchoconstriction) will cause Rrs to increase. In contrast, in emphysema, where there is an increase in compliance, Xrs will decrease, becoming more negative.^69^ In Figure 1 is a graphical representation of the resistance and reactance curves, with all indices highlighted.

**Figure 2.**
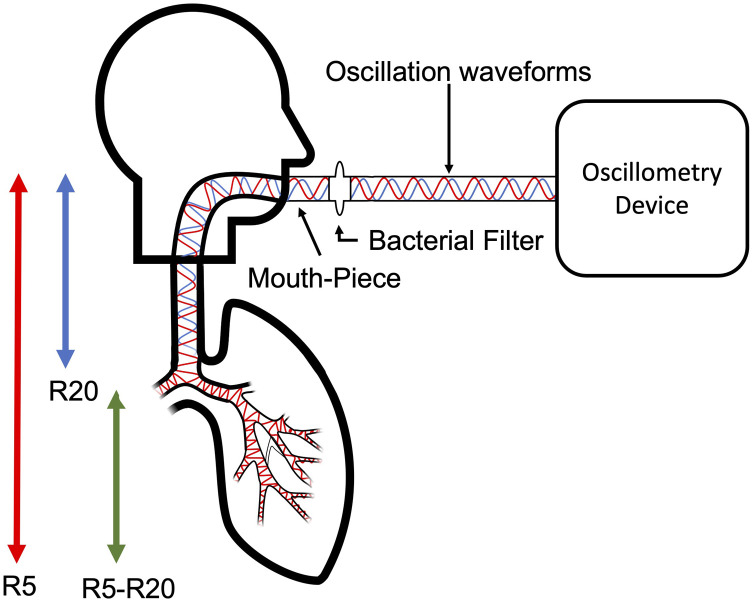
A diagrammatic representation of oscillometry. Legend: Higher frequencies travel shorter than lower frequencies. Resistance at 20 Hz (R20) (blue wave) is considered as a measure of larger airways, and resistance at 5 Hz (R5) (red wave), where waveforms travel further and considered as a measure of the total airways, therefore R5-R20 is considered as a measurement of smaller airways.

The main indices reported in oscillometry are the total airway resistance measured at 5 Hz (R5), large airways resistance, measured at 20 Hz (R20) and small airways resistance (R5-R20), which is simply the difference between R5 and R20, reactance at 5 Hz (X5) and AX, which is the area under the Xrs curve. Figure 2 is an illustration of how waveforms travel and how resistance is assessed.

For patients, oscillation techniques are simple and non-invasive. It involves the patient making a seal around the mouthpiece of the device, holding their cheeks and breathing normally and quietly. When switched on, the device sends oscillatory pressure waves through the mouth into the airways. The test is short, with measurements recorded in under 20 s. The use of tidal breathing makes this measurement particularly useful in patients who have difficulty understanding or performing forced manoeuvres. However, the use of oscillation techniques has been limited by a number of factors. First, there is a need for large population-based studies to produce predicted values for a broader group of patients globally.^70^ Second, different oscillometry devices have been shown to produce different values, limiting the ability to compare results across studies and to form large oscillometry databases that can be used in calculating reference values.^71^ Despite this, there is some evidence that oscillometry can identify different asthma phenotypes,^72^ which makes the test worthy of further investigation and development.

### Oscillometry in asthma

Previous diagnostic test accuracy (DTA) studies have described that R5 had a sensitivity of 69–72% and specificity of 61–86% in diagnosing asthma when a positive bronchodilator response in FEV_1_ was used as a reference standard,^73,74^ comparing favourably to FEV_1_. A systematic review and meta-analysis reviewing the DTA of bronchodilation response (BDR) using FEV_1_ found a sensitivity of 38.9% (95%CI: 18.3–65.6) and specificity of 94.6 (95%CI: 85.7–99.7)^75^. Also, these tests might be able to differentiate whether respiratory symptoms relate to active asthma or another disease process. One study suggested small airways evaluated by IOS indices, R5-R20 and X5 and AX correlated more strongly with clinical symptoms (assessed by the asthma control test (ACT) score) than spirometry.^76^

### Multiple breath washout

**Figure 3.**
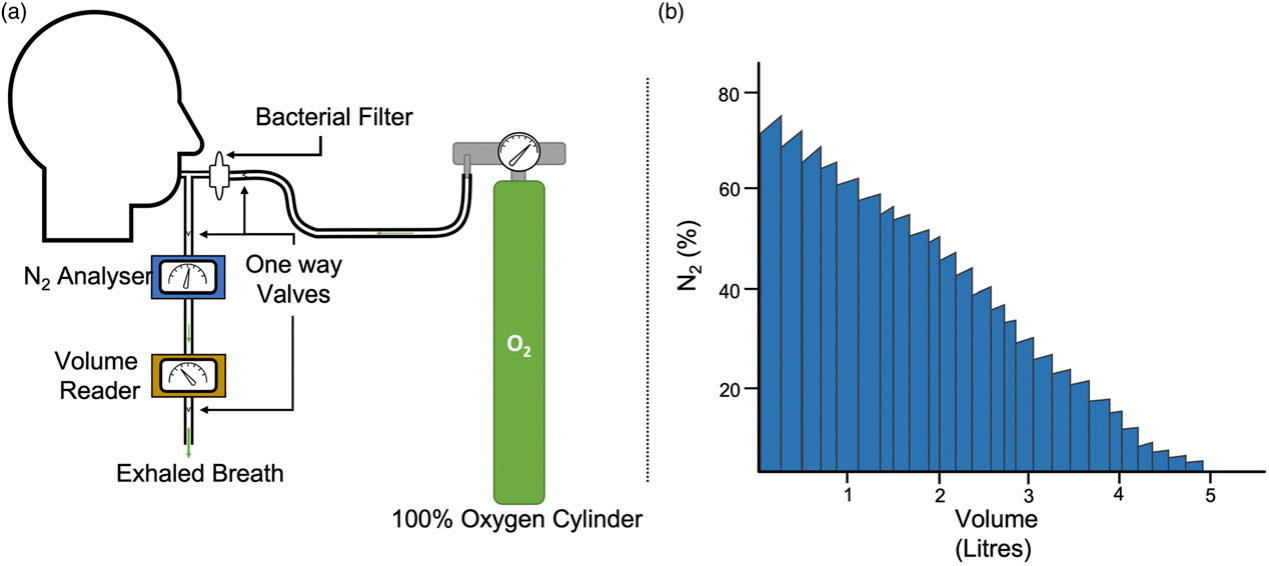
A diagrammatic representation of how intrinsic multiple breath washout is conducted. Legend: In (A) subjects breathe 100% of oxygen to washout nitrogen within the lungs and the amount of exhaled air is calculated. In (B) the amount of nitrogen exhaled nitrogen is analysed and volume is quantified. Lung clearance index is calculated by dividing the cumulative exhaled volume by the functional residual capacity.

Ventilation heterogeneity is defined as the non-uniform distribution of inspired gas within the lung, which can be caused by luminal inflammation, mucus, variable thickening of the airway walls, smooth muscle hyperplasia/hypertrophy, and mucous cell metaplasia. The multiple breath washout (MBW) was first introduced in the 1950s to calculate ventilation heterogeneity using the lung clearance index (LCI). The test can be conducted using two techniques; intrinsic and extrinsic methods. In the intrinsic method, with air within the lungs and airways is ‘washout out’ as the patient breathes 100% oxygen and the measured volume of nitrogen is used to calculate the functional residual capacity (FRC).^77^ . With the extrinsic technique, a tracer gas such is sulphur hexafluoride (SF6) is initially washed in and then washed out of the airways, which may be useful in overcoming the effects of high concentration of oxygen on breathing pattern in some populations.^78^ The MBW test has also been considered in the assessment of small airway function. Although MBW is a simple, submaximal and non-invasive assessment, it is time consuming^79^ and interpretation of the results can sometimes be difficult.^57^ S_acin_ and S_cond_ are used as the two main indices to determine ventilation heterogeneity. The S_acin_ is the ventilation heterogeneity peripheral to the acinar entrance, while S_cond_ is the ventilation heterogeneity at the conductive lung zone.^80^ Figure 3 provides a representation of how MBW is conducted.

### MBW in asthma

There are a number of studies of MBW in asthma, of which only a few discuss or reference SAD. In patients with asthma, an abnormally high S_acin_ has been associated with an increased likelihood of responsiveness to inhaled corticosteroids and was considered suggestive of SAD.^81^ S_cond_ is a predictor of airway hyper-responsiveness in adults with asthma.^82^

Despite the potential of MBW test in assessing small airways function and the advantages of a test which allows tidal breathing, the software for calculating S_acin and_ S_cond_ is not widely available and limited to specialist centres. MBW also lacks robust, universally accepted reference ranges.^77^ Table 2 provides a summary of small airways function tests.

**Table 2.** Summary of advantages and disadvantages of small airways function tests.

Small airways function test	Advantages	Disadvantages/Limitation	Reference range
FOT/IOS	Easy to perform	There are differences among different devices Difficult to interpret due to lack of training Lack of agreed reference ranges	Limited to some populations
MBW	Easy to perform	Relatively time consuming Difficult to interpret	Available
FEF_25-75_ (MMEF) FEF_50_	Highly available Easy to interpret Widely implemented	Patient dependent Highly variable	Available
FeNO	Widely implemented Easy to interpret Easy to perform	Not sensitive to all phenotypes Not conclusive for small airways Not sensitive with patients on ICS^**^	Available

FOT/IOS: forced oscillation technique/impulse oscillometry; MBW: multiple breath washout technique; FEF_25-75_: the mean forced expiratory flow between the 25% and 75% of the forced vital capacity; MMEF: the mean mid-maximal expiratory flow; FEF_50_: The forced expiratory flow in the middle of the forced vital capacity; FeNO: fractional exhaled nitric-oxide; ICS: inhaled corticosteroids.

### Lung volumes

Using body plethysmography or gas dilution techniques, specific lung volumes and capacities can be obtained including residual volume (RV), FRC and total lung capacity (TLC). An increased RV may be linked to small airways obstruction due to air trapping caused by narrowing of the airways. The RV/TLC ratios have been used to assess the presence SAD.^22,32^ Moreover, RV/TLC is considered a more specific index of air trapping in asthma.^57^ Similar to spirometry, lung volume parameters have robust reference ranges for most populations.^83–85^ The disadvantage of lung volumes is mainly around their specificity to small airways obstruction,^57^ as elevated values can be due to other disease processes causing hyperinflation (such as emphysema).

### Lung volumes in asthma

In one study of 321 physician-diagnosed subjects with asthma, 52% and 57% had an abnormal residual volume and abnormal RV/TLC ratio, respectively. There was a negative correlation between RV and FEF_25–75_. The authors described a significant proportion of asthmatic patients having an elevated residual volume and an abnormal RV/TLC ratio in the presence of normal FEV_1_/FVC ratio and absence of significant BDR.^86^ A second study also found a high prevalence of RV dysfunction in asthma patients with a normal FEV_1_/FVC ratio but with symptoms,^87^ highlighting the potential for this test to identify patients with active lung disease.

## The use of small airways in the clinical diagnosis of asthma

The evidence presented suggest that tests of small airways function can be altered in some patients with asthma, but to be adopted into clinical practice, these differences have to have clinical significance in terms of diagnosis, pathology, progression or treatment response.

Head-to-head studies have reported that some patients with symptoms of or inflammation consistent with asthma, have altered small airways test results, even without objective evidence of airflow obstruction (add reference). Since asthma remains a clinical diagnosis, and a patient can have asthma without measurable airflow obstruction at the time of testing, the combination of symptoms and SAD could be sufficient to diagnose asthma, and may help identify specific groups of patients to inform treatment approaches. Studies to date have not shown major differences in the inflammatory endotype of patients with SAD and those without, but SAD is commonly associated with eosinophilic inflammation (add reference), which points towards a specific group of therapies. Ultrafine aerosol particles can reach the small airways, and therefore it would make sense to target these therapies with people with measurable SAD and use SAD to track the response to therapy.

Next to consider is which tests to deploy. There are no gold standard recommended tests of SAD. Of the tests of small airways discussed above, only MMEF has well validated predicted ranges, although these are wide. With other tests, there would be uncertainty as to what represented an abnormal result. MMEF is routinely captured during normal spirometry, and therefore has no extra equipment or resource costs, and does not lengthen the time to perform tests. A disadvantage of MMEF is the forced nature of the manoeuvre. IOS, FOT and MBW use tidal breathing, but MBW is a lengthy test and requires specialised equipment. IOS and FOT also require specific devices, but the test is quick to perform and can be used by the bedside. MBW, IOS and FOT can be difficult to interpret, and with IOS and FOT, the exact device used alters results, meaning results are not comparable across centres.

The ongoing COVID pandemic has significantly impacted the provision of pulmonary function tests, leading to delays in the assessment of pulmonary diseases. Infection control measures in lung function laboratories aim to prevent the transmission of diseases between healthcare providers and patients or participants.^88^ The evidence for the transmission of infections is limited^89^ but pulmonary function testing is considered an aerosol-generating procedure. During the COVID-19 pandemic many laboratories reduced or stopped providing these tests. There is some evidence that FVC manoeuvres produce more and larger droplet particles than tidal breathing^90,91^ and so it might be the case that submaximal assessments such as oscillometry or MBW, may be safer in terms of aerosol generation. However, this would need to be specifically tested.

In summary, these tests appear to be telling us something about certain groups of people with asthma, and the expansion of ultrafine inhaled therapies and strategies to target eosinophilic asthma may provide a clinical rationale to assess small airways. However, there are still barriers to deploying these tests in the clinic.

## Knowledge gaps that limit the utility of small airways testing in asthma

Although studies have aimed to stratify asthma by small airways function, comparing study results is complex. The lack of a gold standard to assess SAD has resulted in studies varying in testing protocols, equipment used, and accepted cut-offs for identifying diseased and non-diseased states. Even within a specific methodology (such as oscillometry), different devices capture different measurements, which has prevented the development of common reference ranges.^71^ These constraints have considerably limited our ability to draw firm conclusions from data published to date, despite promising results from smaller studies.

More research is urgently needed. First, to understand the importance of inflammation within the small airways in asthma and to identify if this requires specific treatment. Second, to clarify which tests of small airways function are most useful in identifying pathological change in asthma. This includes assessing when each test becomes abnormal and to decipher if certain tests are identifying different cohorts or subsets of patients. This will evaluate their diagnostic accuracy to determine if they might be of use in all cases of asthma (which current evidence suggests is unlikely) or a subset of patients (a phenotype). Third, to assess the variability of repeat testing and the accuracy of SAD tests in identifying disease severity and treatment response. Lastly, to determine if there is a relationship between measured SAD and inflammatory signals. If so, this could be used to stratify treatment decisions by inflammatory pathway and degree, potentially improving patient outcomes. However, this approach requires a consensus on the methods for assessing small airways to interpret insights from different studies.

## Conclusion

Asthma is a common and increasingly prevalent condition associated with poor patient outcomes, including mortality and morbidity. There is an ongoing need to adequately diagnose, manage and control asthma more effectively. Asthma is heterogeneous, comprising several clinical phenotypes and cellular endotypes with different responses to treatment. We need specific tools to identify the subsets of patients most likely to benefit from specific and stratified therapeutic approaches and to monitor treatment response.

Studies over time have highlighted the involvement of the small airways in asthma pathology and recent advances in treatments that now reach the small airways offer a new therapeutic paradigm for asthma. Multiple physiological tests of small airways exist, which could be used to identify SAD and measure the response to treatment. However, currently there is no universally accepted approach to choosing a specific test for a specific population or clinical question and there still needs to be significant expansion of reference ranges to aid interpretation. Further research is needed but this is still an area of great promise that could potentially improve patient care and outcomes.
